# Chromosome-scale genome assembly provides insights into the evolution and flavor synthesis of passion fruit (*Passiflora edulis* Sims)

**DOI:** 10.1038/s41438-020-00455-1

**Published:** 2021-01-08

**Authors:** Zhiqiang Xia, Dongmei Huang, Shengkui Zhang, Wenquan Wang, Funing Ma, Bin Wu, Yi Xu, Bingqiang Xu, Di Chen, Meiling Zou, Huanyu Xu, Xincheng Zhou, Rulin Zhan, Shun Song

**Affiliations:** 1 Haikou Experimental Station, Chinese Academy of Tropical Agricultural Sciences, Key Laboratory of Genetic Improvement of Bananas, 571101 Haikou, Hainan P. R. China; 2 Hainan University, 571101 Haikou, Hainan P. R. China; 3 State Key Laboratory of Biobased Material and Green Papermaking, Qilu University of Technology (Shandong Academy of Sciences), 250353 Jinan, Shandong P. R. China; 4 The Institute of Tropical Bioscience and Biotechnology, Chinese Academy of Tropical Agricultural Sciences, 571101 Haikou, Hainan P. R. China

**Keywords:** Genome evolution, DNA sequencing

## Abstract

Passion fruit (*Passiflora edulis* Sims) is an economically valuable fruit that is cultivated in tropical and subtropical regions of the world. Here, we report an ~1341.7 Mb chromosome-scale genome assembly of passion fruit, with 98.91% (~1327.18 Mb) of the assembly assigned to nine pseudochromosomes. The genome includes 23,171 protein-coding genes, and most of the assembled sequences are repetitive sequences, with long-terminal repeats (LTRs) being the most abundant. Phylogenetic analysis revealed that passion fruit diverged after Brassicaceae and before Euphorbiaceae. *Ks* analysis showed that two whole-genome duplication events occurred in passion fruit at 65 MYA and 12 MYA, which may have contributed to its large genome size. An integrated analysis of genomic, transcriptomic, and metabolomic data showed that ‘alpha-linolenic acid metabolism’, ‘metabolic pathways’, and ‘secondary metabolic pathways’ were the main pathways involved in the synthesis of important volatile organic compounds (VOCs) in passion fruit, and this analysis identified some candidate genes, including *GDP-fucose Transporter 1-like*, *Tetratricopeptide repeat protein 33*, *protein NETWORKED 4B isoform X1*, and *Golgin Subfamily A member 6-like protein 22*. In addition, we identified 13 important gene families in fatty acid pathways and eight important gene families in terpene pathways. Gene family analysis showed that the ACX, ADH, ALDH, and HPL gene families, especially *ACX13/14/15/20, ADH13/26/33, ALDH1/4/21*, and *HPL4/6*, were the key genes for ester synthesis, while the TPS gene family, especially *PeTPS2/3/4/24*, was the key gene family for terpene synthesis. This work provides insights into genome evolution and flavor trait biology and offers valuable resources for the improved cultivation of passion fruit.

## Introduction

*Passiflora*, which belongs to the Passifloraceae family in the Malpighiales order, includes more than 520 species worldwide, most of which are distributed in the Americas, including Colombia, Brazil, Ecuador, and Peru, with a few in other tropical and subtropical areas, such as southeast Asia, Australia, and New Zealand^1,2^. Based on morphology, phylogeny, simple sequence repeats (SSRs), and chloroplast genome analysis, it was concluded that the family could be divided into four subgenera: *Astrophaea*, *Decaloba*, *Deidamiodes*, and *Passiflora*^3,4^. In addition, the subgenus *Passiflora* is the largest genus of *Passiflora* and comprises ~240 species that are generally regarded as typical passion flowers. The abundant germplasm resources of the family represent a diversity of phenotypic traits. Some cultivars exhibit good cold tolerance, disease resistance, and fruit quality, providing good materials for genetic breeding and resource development.

*Passiflora* species are widely cultivated in tropical and subtropical areas of the world due to their edible, medicinal, and ornamental value^5,6^. In terms of edible value*, Passiflora* ranks second in the category of edible fruits, with ~60 fruits, including those from *P. edulis*, *P. edulis* f. *flavicarpa*, *P. ligularis*, *P. quadrangularis*, and *P. tarminiana* var. *mollissima*^7^. In terms of medicinal value, many *Passiflora* plants have a long history of use in traditional folk medicines in some American and European countries^8^ as remedies for many neurogenic diseases. The extracts from the leaves, fruits, rind, and seeds have been reported to contain C-glycosyl flavonoids, including vitexin, isovitexin, orientin, isoorientin, luteolin, apigenin, kaempferol, and other active substances with sedative, antioxidant, anti-inflammatory, anxiolytic, and anticarcinogenic effects^9–11^.

In terms of ornamental value, most passion flowers have large floral organs, bright coronal filaments, fine nectariferous structures, a rich fragrance, and luxuriant branches and leaves; thus, they are used as ornamental plants, hedges, and flower racks^12^. Although passion fruit is widely cultivated in the tropical and subtropical areas of the world, Brazil is the largest producer and consumer, with a cropping area of approximately 61,842 ha yielding 923,035 tons per year^13^. In China, passion fruit is planted on 44,466 ha with an annual output of ~590,300 tons and an output value of 3 billion yuan.

To date, genetic research on passion fruit has mainly focused on evaluating its genetic diversity through identifying molecular markers, constructing fingerprints, and determining genetic relationships^13–15^. The cytogenetics of the subgenus *Passiflora* are well established. Most *Passiflora* subgenera are diploid, but the number of chromosomes varies. For example, *P. edulis* and *P. alata* have 2n = 18, while *P. foetida* has 2n = 20^16^. Recently, researchers constructed a large-insert bacterial artificial chromosome (BAC) library and provided the first insight into the structure and content of the passion fruit genome. The library consisted of 82,944 clones with an average insert size of 108 kb^13^. This BAC library provides a new resource for genetic and genomic studies as well as a valuable tool for future whole-genome studies. However, the genome of passion fruit has not been sequenced, and species-specific molecular markers have not yet been developed. The lack of transcriptional and genomic information on passion fruit greatly limits genetic and breeding research on this species; thus, more detailed molecular and genomic resources are needed to thoroughly investigate the genetic diversity of passion fruit species.

Although passion fruit is an economically and ornamentally important tropical fruit, the lack of available genomic information greatly limits the study of passion fruit. In this study, we assembled a high-quality passion fruit genome and performed a detailed analysis of the genome evolution and underlying mechanism of flavor synthesis, which will lay a solid foundation for genetic studies and improvements in passion fruit.

## Materials and methods

### Plant materials, library construction, and sequencing

The plant materials for *P. edulis*, a cultivar of purple passion fruit (Supplementary Fig. 1), were provided by the Haikou Experimental Station (Institute of Tropical Fruit Tree Research), Chinese Academy of Tropical Agricultural Sciences (CATAS). Total genomic DNA was isolated from the fresh leaves of passion fruit using a QIAGEN® Genomic Kit and purified from the gel using a QIAquick Gel Extraction Kit (QIAGEN). A total of 10 μg of high-molecular-weight DNA was sheared using a Megaruptor (Diagenode, NJ, USA). BluePippin (Sage Science, USA) was used to recycle the large DNA fragment. DNA repair (NEBNext FFPE DNA Repair Mix, NEB M6630) and dA tailing (NEBNext Ultra II End-Repair/dA-tailing Module) were then performed. After that, ligation was performed by adding Adaptor Mix (SQK-LSK108 Ligation Sequencing Kit 1D, Oxford Nanopore Technologies). Finally, a 1-μL aliquot was quantified with a Qubit® 3.0 Fluorometer (Invitrogen, USA) to ensure that ≥500 ng of DNA was retained. The purified library was loaded onto flow cells for sequencing on a PromethION (Oxford Nanopore Technologies). Base-calling analysis of unprocessed data was performed using Oxford Nanopore Albacore software (v2.1.3). Two nanopore cells were loaded, and following data quality control, 8.42 million Nanopore reads (~171.4 Gb of data, ~122.43× coverage) were generated using the Nanopore Technologies system. The Nanopore reads had a mean length of 20.35 kb and an N50 length of 30.3 kb, with a maximum length of 214.6 kb (Supplementary Table 1).

For Hi-C sequencing, fresh passion fruit plant tissue was cross-linked with formaldehyde at room temperature for 30 min. Nuclear DNA was digested with DpnII. Sticky end repair was then performed, and free blunt ends were ligated. After ligation, DNA was purified and sheared to 300–700 bp fragments. The DNA fragments were used to construct one Hi-C library and sequenced on an Illumina NovaSeq platform. After filtering adapter contamination and low-quality reads by using fastp (v 0.12.6) with the default parameters, 989.88 million (140.5 Gb, 100× coverage) clean paired-end reads were obtained (Supplementary Table 1).

Total RNA was extracted from a mixed sample including the roots, stems, leaves, flowers, and fruit using the HiPure Plant RNA Kit according to the manufacturer’s instructions (Magen, Guangzhou, China). A total of 3 μg of RNA was used for library preparation with insert sizes of 350 bp, and sequencing was performed on an Illumina NovaSeq 6000. The RNA-seq reads of passion fruit were obtained for gene prediction analysis.

### Genome assembly and quality assessment

Before assembly, we first estimated the genome size of passion fruit. K-mer (k = 17) analysis^17^ was performed on 516.05 million 150 bp paired-end cleaned reads, and the genome size was estimated to be 1395.76 Mb (Supplementary Tables 1 and 2; Supplementary Fig. 2).

Nanopore reads were corrected using NextDenovo (https://github.com/Nextomics/NextDenovo) and then used as input for Smartdenovo (https://github.com/ruanjue/smartdenovo) assembly. The parameters for read correction and assembly were as follows: read_cutoff = 1 k, seed_cutoff = 30 k; -k 21 -J 5000. This resulted in a first assembly with a total size of ~2472.5 Mb and a contig N50 of ~6.3 Mb (Supplementary Table 4). After finishing the initial assembly, iterative polishing was conducted using NextPolish (https://github.com/Nextomics/NextPolish). Polishing was performed three times for the Nanopore reads and four times for the cleaned reads from NovaSeq 6000. The corrected genome (the size was ~2516.5 Mb with a contig N50 of ~6.4 Mb, Supplementary Table 4) was subjected to redundancy elimination using Hi-C technology. Based on the Hi-C data, ~175.69 million valid paired-end reads were used to assist genome assembly (Supplementary Table 3). The genome sequence contigs were divided into subgroups and sorted and oriented into pseudomolecules using LACHESIS^18^ with the following parameters: CLUSTER MIN RE SITES = 100, CLUSTER MAX LINK DENSITY = 2.5, CLUSTER NONINFORMATIVE RATIO = 1.4, ORDER MIN N RES IN TRUNK = 60, and ORDER MIN N RES IN SHREDS = 60. In the end, ~1327.18 Mb contigs (98.92%) were anchored to nine pseudomolecules (Supplementary Table 4).

The accuracy of the Hi-C assembly was evaluated using various methods. We first inspected the Hi-C contact heatmap, and an elevated link frequency was observed with a diagonal pattern within individual pseudochromosomes, indicating increased interaction contacts between adjacent regions (Supplementary Fig. 3). Additionally, we performed BUSCO^19^ (Embryophyta dataset) assessments on the assembly. Approximately 91.56% of the complete gene elements were found in the genome (Supplementary Fig. 4 and Supplementary Table 5). Finally, bwa^20^ and minimap2^21^ were used to compare the second-generation and third-generation data with the corrected genomes to assess their coverage, with mapping rates of 98.74% and 99.99%, respectively (Supplementary Table 6).

### Genome annotation

We identified de novo repetitive sequences in the passion fruit genome using RepeatModeler (v1.0.4) (https://github.com/rmhubley/RepeatModeler) based on a self-BLAST search. We further used RepeatMasker (v4.0.5) (http://www.repeatmasker.org/) to search for known repetitive sequences using a cross-match program with a Repbase-derived RepeatMasker library and the de novo repetitive sequences constructed by RepeatModeler. The repeat-masked genome was used as input to two categories of gene predictors.

Protein-coding genes were predicted using a pipeline that integrated de novo gene prediction and RNA-seq-based gene models. For de novo gene prediction, Augustus (v3.0.3)^22^ and SNAP (v2006-07-28 https://github.com/KorfLab/SNAP) were run with the default parameters, and the training sets used were monocots and maize, respectively. For RNA-seq-based prediction, 6 Gb of RNA-seq reads from a mixed tissue sample were filtered to remove adaptors and trimmed to remove low-quality bases. Processed RNA-seq reads were aligned to the reference genome using TopHat2 (version 2.0.7)^23^. The transcripts were then assembled using Cufflinks (version 2.2.1)^24^.

The rRNAs were predicted using RNAmmer (v1.2)^25^, the tRNAs were predicted using tRNA scan-SE (v1.23)^26^, and other ncRNA sequences were identified using the Perl program Rfam_scan.pl (v1.0.4) by inner calling using Infernal (v1.1.1)^27^.

Functional annotation of the protein-coding genes was carried out by performing BlastP (e-value cutoff 1e-05) searches against entries in both the NCBI Nonredundant (Nr) and Swiss-Prot databases. Searches for gene motifs and domains were performed using InterProScan (v5.28)^28^. The Gene Ontology (GO) terms for genes were obtained from the corresponding InterPro or Pfam entry. Pathway reconstruction was performed using KOBAS (v2.0)^29^ and the Kyoto Encyclopedia of Genes and Genomes (KEGG) database.

### Transcription factor (TF) and protein kinase (PK) annotation

To detect known TFs and PKs in the passion fruit genome, we used the iTAK program^30^ to identify TFs and PKs. The predicted gene sets were then used as queries in searches against the database. Finally, putative TFs belonging to 88 families and putative PKs belonging to 113 families were identified (Supplementary Table 9).

### Construction of phylogenetic trees and estimation of evolution rates

Orthologous gene clusters in passion fruit genomes and 19 other representative plants (Supplementary Table 12) were identified using the OrthoMCL program^31^. A total of 10184 homologous groups containing 21683 genes were identified in passion fruit, and single-copy orthologs were also identified in this set. The single-copy orthologous genes were used to build a maximum likelihood (ML) tree using FastTree (v2.1.9)^32^. This ML tree was converted to an ultrametric time-scaled phylogenetic tree by r8s using the calibrated times from the TimeTree^33^ website.

### Syntenic and *Ks* analysis

Syntenic blocks were identified using MCScanX with the default parameters^34^. Proteins were used as queries in searches against the genomes of other plant species to find the best matching pairs. Each aligned block represented an orthologous pair derived from the common ancestor. *Ks* (the number of synonymous substitutions per synonymous site) values of the homologs within collinear blocks were calculated using the Nei-Gojobori approach implemented in PAML^35^, and the median *Ks* value was considered to be representative of the collinear blocks. The values of all gene pairs were plotted to identify putative whole-genome duplication events within passion fruit. The duplication time was estimated using the formula t = *Ks*/2r, which represented the neutral substitution rate and was used to estimate the divergence time between passion fruit and other species. A neutral substitution rate of 8.12 × 10^−9^ was used in the current study.

### Metabolome analysis

Three fruit samples (T1, 2 weeks before harvest; T2, at harvest time; T3, 1 week after harvest) with six biological replicates were collected to extract metabolites (Supplementary Table 10). One gram of the powder was transferred immediately to a 20 mL headspace vial (Agilent, Palo Alto, CA, USA) containing 2 mL of NaCl saturated solution to inhibit any enzyme reaction. Fully automatic headspace-solid phase microextraction (HS-SPME) was used for sample extraction for gas chromatography-mass spectrometry (GC-MS) analysis. The parameters for SPME were aging temperature: 250 °C; aging time: 5 min; heating temperature: 60 °C; heating time: 10 min; adsorption time: 20 min; analysis time: 5 min; and aging time after injection: 5 min.

Qualitative analysis of the raw data obtained via GC-MS was performed using Qualitative Analysis Workflows B.08.00 software, and quantitative analysis was performed using MassHunter software. The internal standard was used to normalize the quantitative data. Metabolites with significant differences in content, indicated by a fold-change ≥2 or ≤0.5, a *P* value <0.05, and variable importance in project (VIP) ≥ 1, were considered significantly differential metabolites. The identified metabolites were annotated using the KEGG compound database (http://www.kegg.jp/kegg/compound/), and the annotated metabolites were then mapped to the KEGG pathway database (http://www.kegg.jp/kegg/pathway.html).

### Transcriptome sequencing

All three samples (T1, T2, and T3) were frozen in liquid nitrogen immediately following harvesting. Each sample had two biological replicates. RNA isolation, library construction, and sequencing were performed as described for the RNA-seq analysis for gene prediction analysis. Raw reads were trimmed to remove adaptors and enhance quality. Reads that were <100 bp in size after trimming were discarded. Overall, 94,885,772 (two replicates) to 98,420,318 (two replicates) raw reads were obtained for each sample (Supplementary Table 10).

The TopHat2 package (version 2.0.7)^23^ was used to map clean reads to the genome with the default parameters. Transcripts were assembled using Cufflinks (version 2.2.1)^24^. Gene expression was measured as fragments per kilobase of transcript per million fragments mapped (FPKM) using Cufflinks. Differentially expressed genes (DEGs) were determined using DEseq2^36^. The false discovery rate was used to adjust the *P*-values. Genes with significant differences in expression, |log2Fold Change | ≥ 1, and adjusted *P*-value <0.05 were considered DEGs and were annotated to GO terms and KEGG pathways.

### Functional gene analysis

To investigate the genes involved in the volatile organic compound (VOC) biosynthesis pathways, especially the fatty acid and terpene metabolic pathways, in the passion fruit genome, 21 gene families were detected from the HMM domain models and BLASTP results. Terpenoid synthase (TPS) proteins, alcohol acetyltransferase (AAT) proteins, alcohol dehydrogenase (ADH) proteins, aldehyde dehydrogenase (ALDH) proteins, and lipoxygenase (LOX) proteins were identified by screening the passion fruit genome sequences using HMMER3.0 software with domain models PF03936 and PF01397; PF07247; PF08240, and PF00107; PF00171; and PF00305 as queries^37^. Hydroperoxide lyase (HPL), acyl carrier protein-acyltransferase (ACP-AT), acyl-CoA oxidase (ACX), pyruvate decarboxylase (PDC), cytochrome P450 (CYP), epoxyde hydrolase (EHL), fatty acid desaturase (FAD), fatty acid epoxidase (FAE), fatty acid hydroxylase (FAH), hydroperoxidase lyase (HPL), isopentenyl-diphosphate δ-isomerase (IDI), monoterpene synthase (MTS), carotenoid cleavage dioxygenase (CCD), farnesyl diphosphate synthase (FPS), geranylgeranyl diphosphate synthase (GGPS), geranyl-diphosphate synthase (GPS), and 4-hydroxy-3-methylbut-2-enyl diphosphate reductase (HDR) proteins were identified by BLASTP based on *Arabidopsis thaliana*-related proteins. We then confirmed the presence of the conserved domain within all protein sequences and removed members without a complete domain. The protein domains of these homologs were predicted by Pfam (http://pfam.xfam.org/). Only the genes with the same protein domain were considered homologs. The cis-element analysis of the ADH and TPS gene family promoters was carried out using the cis-acting regulatory elements database (PLANTCARE) (http://bioinformatics.psb.ugent.be/webtools/plantcare/html/).

## Results

### Genome sequencing and assembly

**Fig. 1:**
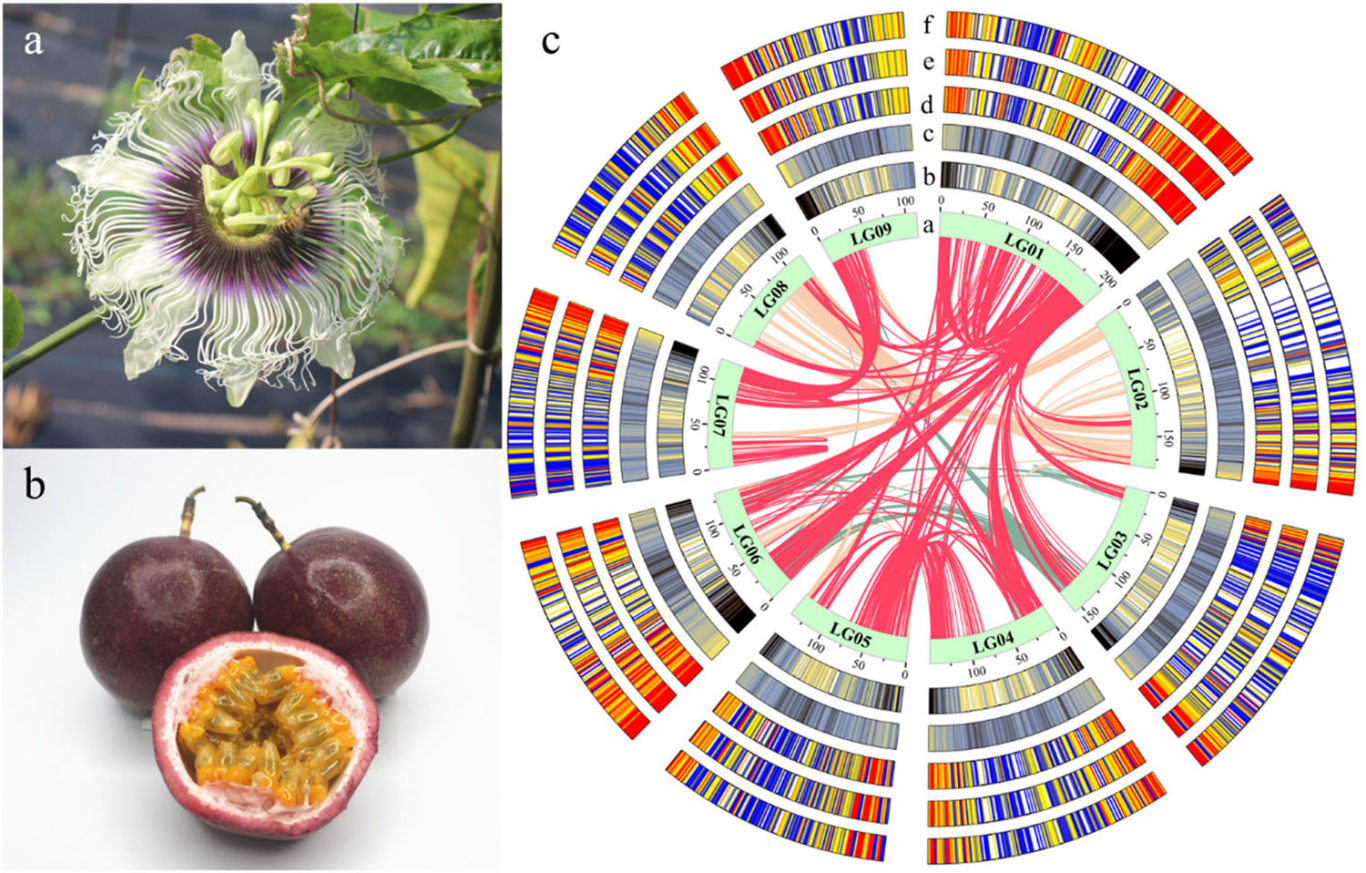
Morphology of passion fruit and overview of the passion fruit genome. **a** Flower of *P. edulis* purple passion fruit cultivar. **b** Fruits of *P. edulis* purple passion fruit cultivar. **c** Elements are arranged in the following scheme (from inner to outer). **a** The nine chromosomal pseudomolecules: units on the circumference are mega base values of pseudomolecules; **b** gene density; **c** repeat density and gene expression levels in three stages of fruit development: **d** T1; **e** T2; and **f** T3. **b**, **c** are shown in 1-Mb windows with a 200kb sliding window, and (**d, e, f**) are shown in 50-kb windows with a 5-kb sliding window. Central colored lines represent syntenic links between the chromosomes

The *P. edulis* purple passion fruit cultivar, with a large and attractive corolla (Fig. 1a), purple skin and yellow pulp (Fig. 1b), is the most widely planted cultivar in the world; thus, we selected it for sequencing and assembly. We estimated the genome size of purple passion fruit as 1395.76 Mb by K-mer analysis (Supplementary Table 2 and Supplementary Fig. 1). A total of 8.42 million Nanopore clean reads (~171.4 Gb data, ~122.43× coverage) were generated using the Nanopore Technologies system (Supplementary Table 1). After genome assembly, polishing, and redundancy elimination, the final assembly size of the genome was ~1341.7 Mb with a contig N50 of ~3.1 Mb (Supplementary Table 4). To improve the quality of the genome assembly and anchor the contigs to chromosomes, we constructed high-throughput chromosome conformation capture (Hi-C) libraries of passion fruit, generating 140.5 Gb (100.36×) of Hi-C paired-end reads (Supplementary Tables 1 and 3). Duplicate removal, sorting, and quality assessment were performed with Hi-C-Pro, and uniquely mapped valid reads were used for Hi-C scaffolding (Supplementary Fig. 3). As a result, 1327.18 Mb (98.92%) of the assembly, and 22,261 (96.07%) of the genes were placed on nine chromosomes (Supplementary Fig. 3 and Supplementary Table 4).

We assessed the completeness and accuracy of the assembly using various approaches. First, more than 91.56% of BUSCOs^19^ were found in the genome assembly (Supplementary Fig. 4 and Supplementary Table 5). Second, approximately 84.85% of the RNA-seq data from different stages of fruit development matched the genome assembly (Supplementary Table 10). Third, the assembled sequences were compared with the second-generation and third-generation data, and the mapped rates and coverage ratios were 98.74% and 95.47%, respectively, and 99.99% and 99.95%, respectively (Supplementary Table 6).

### Genome annotation

We annotated the genome using the AUGUSTUS pipeline^22^ incorporating ab initio predictions and transcriptome data from three samples, resulting in 23,171 protein-coding genes in the passion fruit genome (Supplementary Table 4). The number of genes identified was smaller than that in other closely related species, such as *Populus trichocarpa*, *Ricinus communi*s, and *Theobroma cacao*, which have 51,717, 28,584, and 30,854 genes, respectively^38–40^. Of the 23,171 genes, 22,261 (96.1%) were assigned to a chromosomal location (Supplementary Tables 4,7). These genes were unevenly distributed along the chromosomes with a distinct preference for the ends (Fig. 1c). The average gene length (3239 bp) and coding sequence length (204 bp with 5.69 exons) were similar to those of other plant species^39^. The average GC content of passion fruit (38.68%) was lower than that reported in a previous study (42%)^13^ (Supplementary Table 4). We also annotated 213 ribosomal RNAs (rRNAs), 2232 transfer RNAs (tRNAs), and 71 small nuclear RNAs (snRNAs) (Supplementary Table 4).

In total, 994,414 repeat elements were identified in the passion fruit genome assembly, indicating that most of the assembled genome was repetitive (Supplementary Tables 4,7). These repeat elements were unevenly distributed along the chromosomes, with a distinct preference for the centromeres (Fig. 1c). Consistent with the patterns in many other plant genomes, long-terminal repeat (LTR) retrotransposons were the most abundant class of repetitive DNA, with Gypsy repeats being most abundant, followed by Copia (Supplementary Table 8).

Additionally, we identified 1578 TFs among 88 families in the passion fruit genome using the iTAK programmer, representing 6.81% of genes (Supplementary Table 9). The gene families with the most members in the passion fruit genome included bHLH, MYB, ERF, and bZIP (Supplementary Table 9). In addition, a total of 773 PKs among 113 families in the passion fruit genome were identified, of which 438 PKs belong to the RLK-Pelle group (Supplementary Table 9).

### Phylogenetic evolution, whole-genome duplication, and genome synteny

**Fig. 2:**
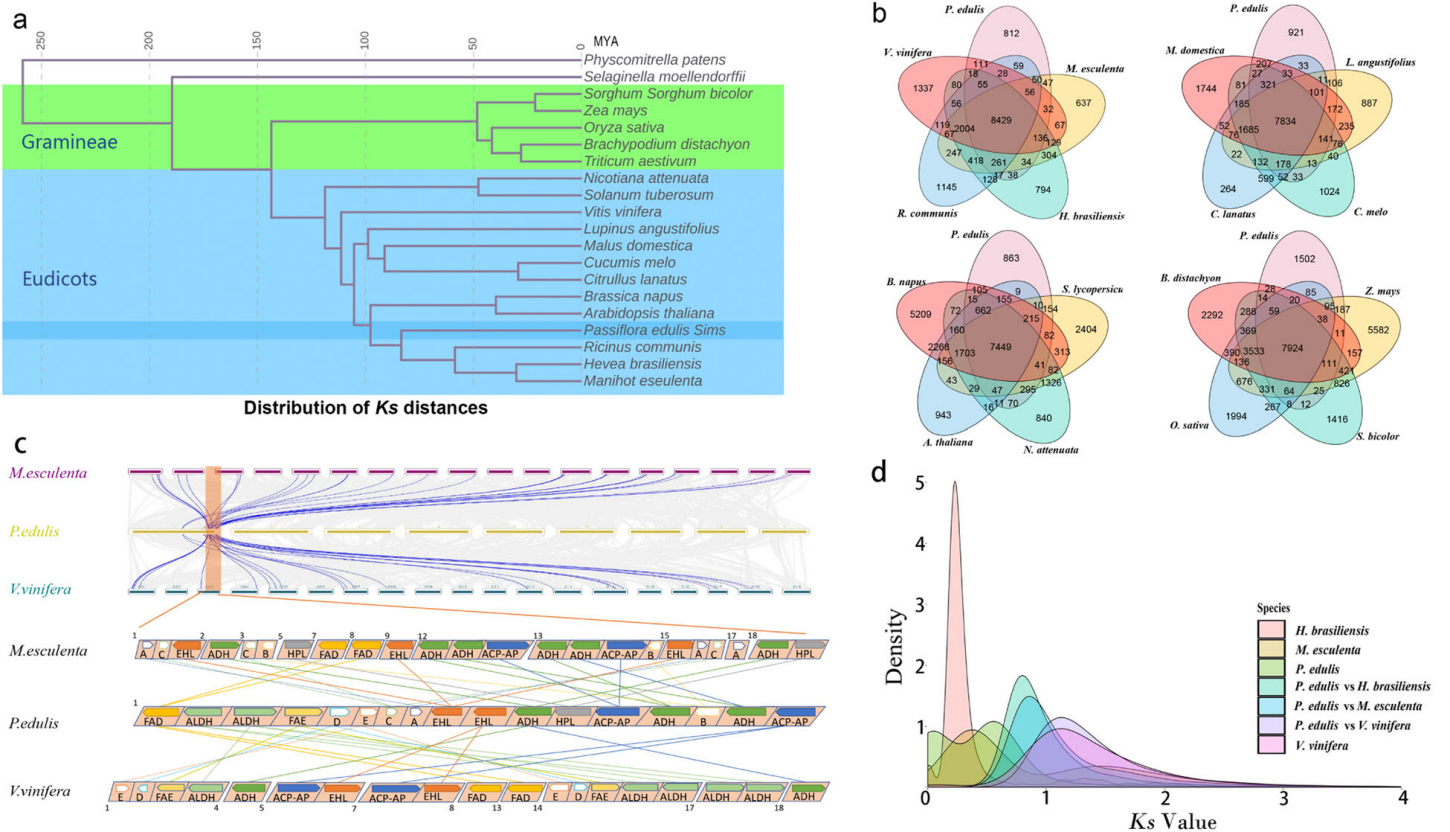
Evolution of the passion fruit genome. **a** Phylogenetic tree of 19 species and *P. edulis*. Inferred divergence times (million years ago) are denoted at each node. **b** Shared and unique gene families in passion fruit and other species. **c** Syntenic blocks between *P. edulis* and *M. esculenta*, *V. vinifera* (top) and syntenic analysis of important genes in a block (bottom). **d** Distribution of the *Ks* values of the best reciprocal BLASTP hits in the genomes of *P. edulis*, *M. esculenta*, *V. vinifera*, *H. brasiliensis*, *P. edulis* vs *M. esculenta*, *P. edulis* vs *V. vinifera*, and *P. edulis* vs *H. brasiliensis*

We identified unique and shared gene families among passion fruit and 19 other species using OthoMCL^31^ (Supplementary Tables 11 and 12): *P. edulis*, *M. esculenta*, *V. vinifera*, *R. communis*, and *H. brasiliensis* share 8429 gene families; *P. edulis*, *L. angustifolius*, *C. lanatus*, *C. melo*, and *M. domestica* share 7834 gene families; *P. edulis*, *S. lycopersicum*, *B. napus, A. thaliana*, and *N. attenuata* share 7449 gene families; and *P. edulis*, *B. distachyon*, *O. sativa*, *Z. mays*, and *S. bicolor* share 7924 gene families (Fig. 2b). We also found that 4247 gene families were shared in all species, while 368 gene families were specific to passion fruit (Supplementary Table 12). GO analysis showed that the terms ‘cell’, ‘cell part’, ‘intracellular’, ‘binding’, and ‘cellular process’ were the most significantly enriched in the specific gene families (Supplementary Fig. 5). Expression analysis showed that most members of the endemic gene family showed low expression levels at different stages of fruit development. Approximately 87 genes showed high expression levels (FPKM > 20) that changed with fruit development (Supplementary Fig. 6).

A phylogenetic tree was constructed using a set of single-copy gene families, and it indicated that passion fruit had the closest relationship with Euphorbiaceae (*R. communis, H. brasiliensis*, and *M. esculenta*) followed by Brassicaceae (*B. Napus* and *A. thaliana*) and was distant from other plants (Fig. 2a). In addition, passion plants evolved independently after separation from the common ancestor of Euphorbiaceae 80 million years ago (MYA). This phylogenetic tree is consistent with the species relationships observed in previous studies^41^.

To clarify when passion fruit underwent whole-genome duplication, synonymous substitutions (*Ks*) were characterized in *V. vinifera, H. brasiliensis, M. esculenta*, and *P. edulis*. The peak *Ks* was 0.6 for homologous gene pairs (Fig. 2b), suggesting that a whole-genome duplication (WGD) event had occurred in passion fruit. The WGD event was estimated to have occurred ~65 MYA (Fig. 2b), indicating that the WGD event occurred after the divergence of passion fruit and Euphorbiaceae (Fig. 2b). In contrast, the peak *Ks* was 0.1 for homologous gene pairs of the passion fruit genome, which indicated another WGD event that was estimated to have occurred ~12 MYA ago. This result was consistent with the phylogenetic tree.

Intergenomic analysis revealed that there were different linear relationships between the chromosomes of the passion fruit genome, and a total of 5545 collinear gene pairs were identified (Fig. 1c). Intergenomic analyses between *P. edulis* and *V. vinifera* and *M. esculenta* revealed highly conserved collinearity, which supports a close evolutionary relationship among these plants. All nine chromosomes in *P. edulis* corresponded strongly to the 18 *M. esculenta* chromosomes, followed by 19 *V. vinifera* chromosomes (Fig. 2c). In total, we identified 14,467 and 21,210 collinear genes between *P. edulis* and *V. vinifera* and between *P. edulis* and *M. esculenta*, respectively, indicating that 62% and 91% of the passion fruit genome was colinear with these plants. In addition, in some important genes (ADH, FAD, and EHL), we can see the collinearity of these three species (Fig. 2c). The results further suggested possible chromosome fusions in the species ancestral to passion fruit or chromosome divergence in the ancestral species.

### Metabolic differences among passion fruit developmental stages

**Fig. 3:**
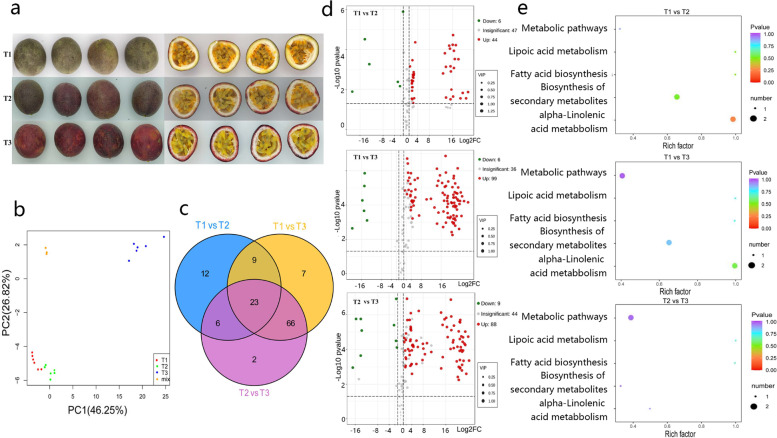
Metabolome analysis during fruit development in *P. edulis*. **a** Representative images of passion fruit at different developmental stages: T1 (2 weeks before harvest), T2 (at harvest time), and T3 (1 week after harvest). **b** Principal component analysis (PCA) of samples with six biological repetitions at three developmental stages. **c** Venn diagram of differential metabolites for T1 vs. T2, T1 vs. T3, and T2 vs. T3. Nonoverlapping regions represent the metabolites that are specific to the different subgroups, while overlapping regions represent the metabolites that are common to several different subgroups. **d** Volcano plot of differential metabolites for T1 vs. T2, T1 vs. T3, and T2 vs. T3. Each point in the figure represents a metabolite. Green points represent downregulated metabolites, red points represent upregulated metabolites, and gray points represent metabolites that were detected but not significantly different. **e** Pathway enrichment analysis of differential metabolites for T1 vs. T2, T1 vs. T3, and T2 vs. T3. The color of the point represents the *P* value, and the size of the point represents the number of differentially enriched metabolites

To understand the molecular mechanisms underlying the differences in VOCs during fruit development, fruits of three different developmental stages were collected (Fig. 3a and Supplementary Table 10). Metabolic analysis revealed 142 metabolites in this study, including esters, terpenes, acids, and alcohols (Supplementary Table 13). To compare the metabolite composition involved in the fruit development of passion fruit, GC-MS data were subjected to principal component analysis (PCA). The metabolite compositions in the three samples were clearly separated in the PC1 × PC2 score plots. The first principal component (PC1) and PC2 were clearly separated between the T1, T2, and T3 samples (Fig. 3b). The PCA readily discriminated T1, T2, and T3, with PC1 and PC2 explaining 46.25% and 26.82% of the total variance, respectively.

### Differential accumulation of flavor substances between T1, T2, and T3

The flavor quality of fruit mainly involves sugars and organic acids involved in carbohydrate metabolism. The contents and types of different metabolites may play a crucial role in determining flavor in passion fruit. The three samples showed differences in the accumulation of sugars, organic acids, and esters. We identified a total of 50, 105, and 97 differential metabolites between T1 and T2, between T1 and T3, and between T2 and T3, respectively (Fig. 3c). Overall, most metabolites had higher levels in T3 than in T1 and T2 and higher levels in T2 than in T1 (Fig. 3c). Between T2 and T1, the content of 44 metabolites increased, whereas that of six metabolites decreased (Supplementary Table 14); between T1 and T3, the content of 99 metabolites increased, whereas that of six metabolites decreased (Supplementary Table 15); and between T2 and T3, the content of 88 metabolites increased, whereas that of nine metabolites decreased (Fig. 3d and Supplementary Table 16). The differential metabolites were annotated using the KEGG database, and the results showed that these pathways were mainly enriched in the following: ‘metabolic pathways’, ‘lipoic acid metabolism’, ‘fatty acid biosynthesis’, ‘biosynthesis of secondary metabolites’, and ‘alpha-linolenic acid metabolism’ (Fig. 3e).

### Transcriptome sequencing, clustering, and functional enrichment of DEGs

**Fig. 4:**
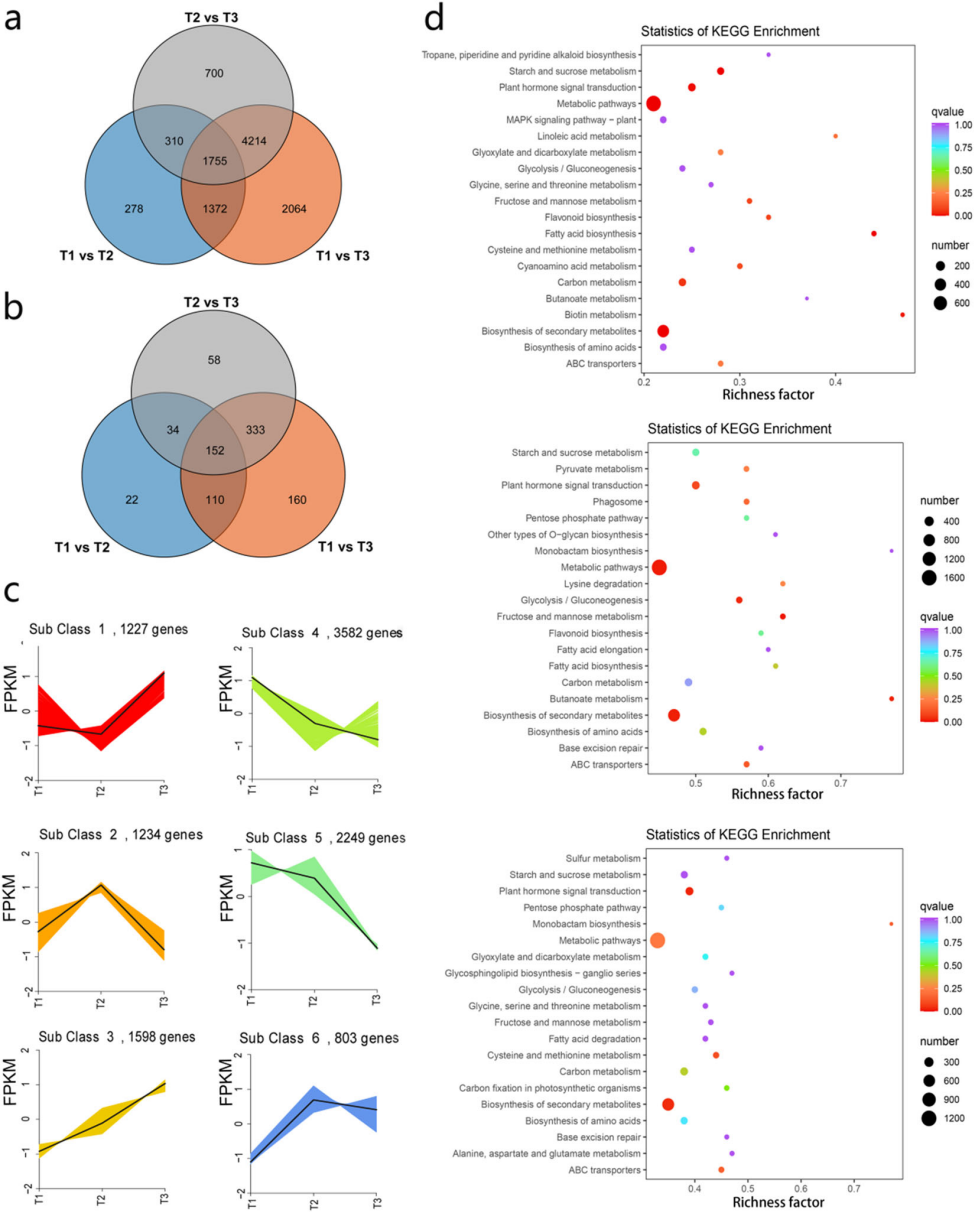
Gene expression patterns and KEGG enrichment analyses during the development of *P. edulis*. **a** Venn diagram of DEGs for T1 vs. T2, T1 vs. T3, and T2 vs. T3. **b** Venn diagram of differentially expressed TFs for T1 vs. T2, T1 vs. T3, and T2 vs. T3. **c** K-means clustering analysis of gene expression patterns in passion fruit. **d** Pathway enrichment analysis of DEGs for T1 vs. T2, T1 vs. T3, and T2 vs. T3. The color of the point represents the *P* value, and the size of the point represents the number of enriched DEGs

Transcriptomic sequencing was performed on the three developmental stages of passion fruit (Supplementary Table 10). After original data filtration, error rate examination, and GC content distribution, we obtained 6.84–7.45 million high-quality 150-bp paired-end reads (Supplementary Table 10). High correlations were obtained for two replications of these samples (Supplementary Figs. 7 and 8). The clean reads were then mapped to the assembled genome, and ~85% of the reads were mapped to the genome, resulting in the annotation of more than 19,224 genes. A total of 10693 DEGs were identified using DESeq2^36^ based on |log2Fold Change | ≥ 1 and FDR < 0.05 in all samples. Among them, 3715 DEGs (1571 upregulated and 2144 downregulated) were identified between the transcriptomes of the T1 and T2 samples (Supplementary Fig. 9), 9405 DEGs (3405 upregulated and 6000 downregulated) were identified between the T1 and T3 samples (Supplementary Fig. 9), and 6979 DEGs (2259 upregulated and 4720 downregulated) were identified between the T2 and T3 samples (Supplementary Fig. 9). We also found 1755 common DEGs and 152 common differentially expressed TFs among the three groups (Fig. 4a, b).

To analyze DEG expression patterns, the expression data at different stages were centralized and standardized and then clustered using K-means. The DEGs in the fruit at the three stages were clustered into six subclasses based on gene expression patterns. The DEGs in subclasses 4 and 5 were significantly enriched in the three samples (Fig. 4c). The DEGs in subclasses 1 and 3 were upregulated, while the DEGs in subclasses 4 and 5 were downregulated in the three samples. A total of 55 enriched GO terms were found, and the terms ‘cell wall organization’, ‘polysaccharide metabolic process’, and ‘hydrolase activity’ were the most significantly enriched between T1 and T2 (Supplementary Table 17). Between T1 and T3, the terms ‘cell wall organization’, ‘cellular polysaccharide metabolic process’, and ‘plasma membrane part’ were the most enriched (Supplementary Table 18). Additionally, the terms ‘cell wall organization’, ‘plasma membrane part’, and ‘cellular carbohydrate metabolic process’ were the most significantly enriched between T2 and T3 (Supplementary Fig. 10 and Supplementary Table 19).

We also used KEGG to enrich the DEGs. Between T1 and T2, the terms ‘metabolic pathways’ (776 DEGs), ‘biosynthesis of secondary metabolites’ (424 DEGs), ‘starch and sucrose metabolism’ (90 DEGs), and ‘fatty acid biosynthesis’ (28 DEGs) were significantly enriched (q-value < 0.05, Supplementary Table 20). The terms ‘fructose and mannose metabolism’ (62 DEGs), ‘metabolic pathways’ (1676 DEGs), and ‘biosynthesis of secondary metabolites’ (886 DEGs) were significantly enriched between T1 and T3 (Supplementary Table 21). In contrast, between T1 and T2, two pathways, ‘plant hormone signal transduction’ (179 DEGs) and ‘biosynthesis of secondary metabolites’ (664 DEGs), were significantly enriched (Fig. 4d and Supplementary Table 22). These pathways provide insights into the metabolic processes underlying different fruit development stages in passion fruit.

### Integrated transcriptomic and metabonomic analysis

PCA of the transcriptomic and metabolomic data from fruit at different stages showed significant differences among the samples (Fig. 3b and Supplementary Fig. 8). KEGG enrichment analysis of differential metabolites and DEGs was performed simultaneously, and the results showed that in the three comparisons (T1 vs. T2, T1 vs. T3, and T2 vs. T3), the significantly enriched pathways were ‘alpha-linolenic acid metabolism’ (T1 vs. T2: 25 DEGs and 1 meta; T1 vs. T3: 60 DEGs and 1 meta; T2 vs. T3: 46 DEGs and 1 meta), ‘metabolic pathways’ (T1 vs. T3: 1676 DEGs and 1 meta; T2 vs. T3: 1236 DEGs and 2 meta), and ‘biosynthesis of secondary metabolites’ (T1 vs. T2: 424 DEGs and 1 meta; T1 vs. T3: 886 DEGs and 1 meta; T2 vs. T3: 664 DEGs and 1 meta; Supplementary Fig. 11). Pearson’s correlation coefficients (PCCs) of the DEGs and differential metabolites in each group were calculated using the ‘Cor’ package in R (https://www.r-project.org/), and the differential multiples of the metabolites with PCC > 0.8 in each group are shown in a nine-quadrant diagram (Supplementary Fig. 12). A total of 2908 DEGs and 61 metabolites for T1 vs. T2, 7753 DEGs and 129 metabolites for T1 vs. T3, and 6593 DEGs and 112 metabolites for T2 vs. T3 were detected in the third and seventh quadrants. The differential patterns of gene expression and metabolites were consistent, suggesting that the metabolite changes may be positively regulated by genes. In addition, differential correlation analysis (PCC > 0.8) showed that 3715 DEGs were related to 23 metabolites for T1 vs. T2, 9406 DEGs were associated with 54 metabolites for T1 vs. T3, and 3715 DEGs were associated with 49 metabolites for T2 vs. T3 (Supplementary Fig. 13).

All the DEGs and differential metabolites were used to establish a two-way orthogonal partial least squares (O2PLS) model, the variables with high correlations and weights in different datasets were preliminarily assessed based on the loading diagram, and the important variables affecting another omics dataset were screened out. The results showed that the top 10 metabolites affected by the transcriptome included three ketones, three esters, two benzenes, one alcohol, and one imine (Supplementary Fig. 14). Additionally, the top 30 genes that were significantly affected by the metabolome are shown in Supplementary Figs. 12 and 14. The expression levels of these genes were largely high in the T2 stage, and the expression levels of nine genes (*GDP-fucose transporter 1-like*, *tetratricopeptide repeat protein 33*, *protein NETWORKED 4B isoform X1*, *golgin subfamily A member 6-like protein 22*, and five unknown functional genes) were high in all three stages (Supplementary Fig. 15).

### Ester and terpene synthesis pathways during fruit development in passion fruits

**Fig. 5:**
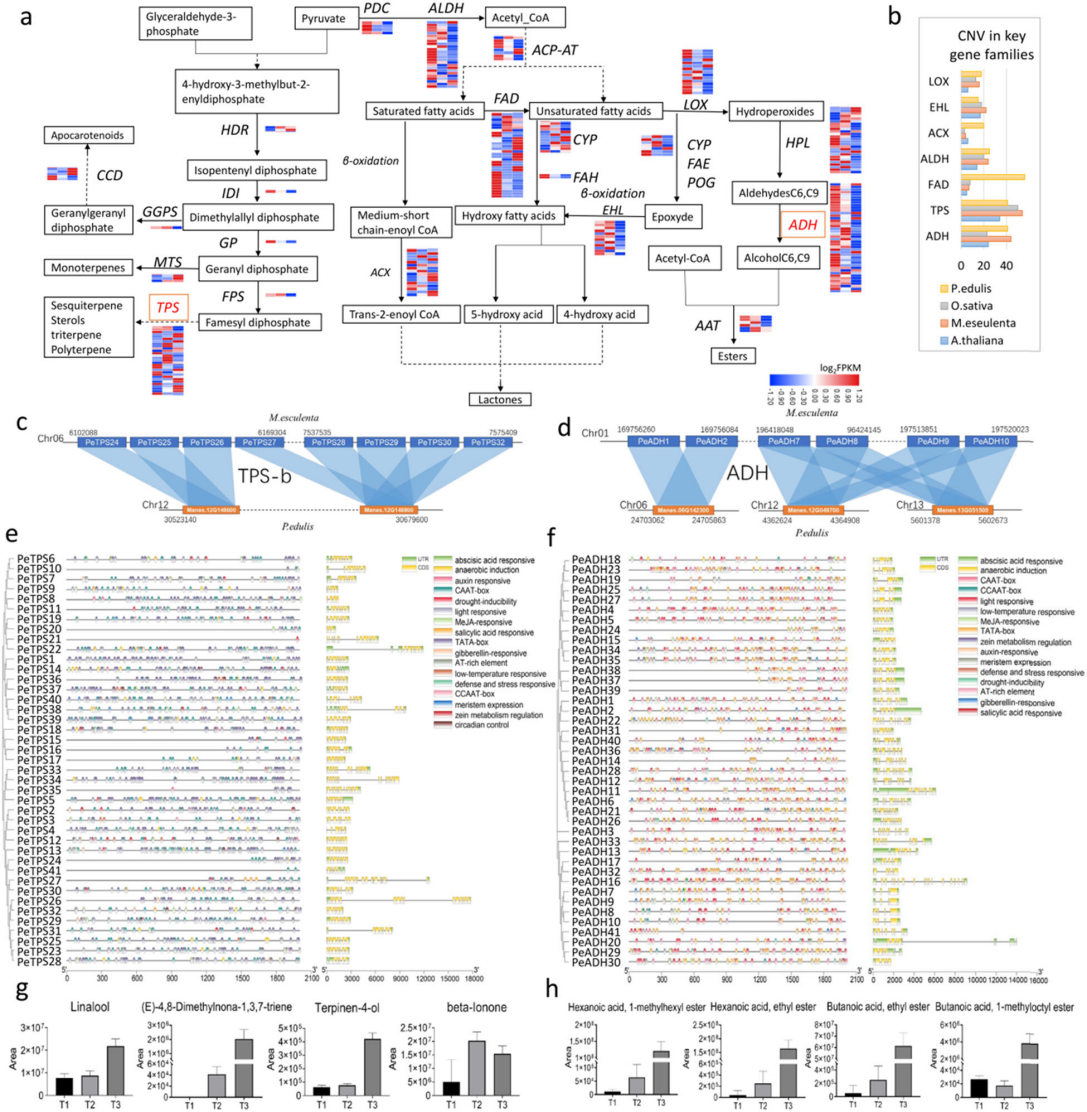
Expression heatmap and metabolite contents of fatty acid metabolic and terpene biosynthesis pathways in passion fruit. **a** Overview of the fatty acid metabolic and terpene biosynthesis pathways showing the gene number expansion at each step and their expression profiles in different stages of fruit development. The heatmap was drawn using log2-based FPKM fold values. Red represents high expression, and blue represents low expression. **b** Copy number variation (CNV) of important gene families in cassava, rice, Arabidopsis, and passion fruit. **c** Tandem repeat gene analysis of the PeTPS gene family. **d** Tandem repeat gene analysis of the PeADH gene family. **e** Summary of major cis-elements in the promoter region of the PeTPS gene family. **f** Summary of major cis-elements in the promoter region of the PeADH gene family. **g** Changes in terpene metabolite contents. **h** Changes in ester metabolite contents

Plant VOCs are secondary metabolites that play an important role in flavor in fruits^42^. In passion fruit, VOCs mainly include esters, alcohols, terpenes, aldehydes, and acids. In the metabolomic analysis, 142 volatile compounds were detected, 63 of which were shared among the three samples. These compounds showed fold changes between 0.23 and 8.8 for T2 vs. T1 and between 0.21 and 10437 for T3 vs. T2 and T3 vs. T1, indicating that the levels of many volatile compounds increased sharply during T3. Among the upregulated metabolites, four compounds increased by more than 100-fold in T3 vs. T1, namely, hexanoic acid 1-methylhexyl ester (10437-fold), hexanoic acid ethyl ester (3896-fold), butanoic acid ethyl ester (1154-fold), and butanoic acid 1-methylloctyl ester (144-fold) (Fig. 5h), all of which are esters that are synthesized by fatty acid metabolic pathways. In addition, 26 compounds were present in only T2 and T3 (12 of which were esters), and 44 compounds were detected in only T3, among which 22 were esters (accounting for half of the total number of compounds in the T3 sample). These results indicated that esters were the main aromatic components of passion fruit. In addition, among the detected metabolites, some terpenes accounted for a large peak area and changed significantly among the three test samples, including linalool, (E) - 4,8-dimethylona-1,3,7-triene 3-buten-2-one, terpinen-4-ol, and 4- (2,6,6-trimethyl-1-cyclohexen-1-yl) (also known as beta-ionone) (Fig. 5g). Therefore, we conclude that terpenes are also important components of passion fruit VOCs. Thus, the key genes for ester and terpene synthesis were identified here.

Esters are mainly synthesized by the fatty acid pathway, and we identified 13 important gene families in this pathway (Fig. 5a). Most of the genes are located on chromosomes 1, 5, and 6 (Supplementary Table 23). We found that the ACX, ADH, ALDH, FAD, and HPL gene families had large numbers of members: 20, 41, 25, 56, and 33, respectively. In addition, compared to Arabidopsis, cassava, and rice, these genes may have undergone gene expansion that enhanced the ability to produce esters (Fig. 5b), and three tandem repeat blocks were also found in the ADH gene family (Fig. 5d). On the other hand, we found that many stress-related and defense-related cis-elements were in the promoter region of the ADH gene family (Fig. 5f). We also constructed a phylogenetic tree by aligning the ADH proteins of passion and *Arabidopsis thaliana* to confirm the classification of ADH family proteins in passion fruit (Supplementary Fig. 16). We elucidated the functions of ADH members of passion fruit and ADH members of *Arabidopsis thaliana* in the same subgroup. Expression analysis showed that the expression levels of *PeACX13/14/15/20*, *PeADH13/26/33*, *PeALDH1/4/21*, and *PeHPL4/6* increased with the development of the fruit, which is consistent with the increasing trend of ester metabolite contents (Fig. 5h), while the level of *PeFAD13/50/52/53/55* gradually decreased. At the same time, other gene families were also represented, including AAT (six proteins), ACP-AP (eight proteins), PDC (four proteins), CYP (16 proteins), EHL (15 proteins), FAE (eight proteins), FAH (two proteins), and LOX (18 proteins). Expression analysis also indicated that some genes changed with fruit development, such as *PeLOX5/18*, *PePDC3*, *PeFAE6*, *PeFAH2*, *PeCYP7/13*, *PeEHL13/15*, *PeACP-AP1/5/6/7*, and *PeAAT3*. These genes, especially the ADH genes, may play an important role in the metabolism of esters.

Terpene metabolites are mainly produced by the terpene metabolic pathway, and we identified eight important gene families in this pathway (Fig. 5a). Most of the genes were located on chromosomes 4, 6, and 8 (Supplementary Table 23). The family with the largest number of members was the TPS gene family, with 41 members. The amount of TPS, which may play a key role in terpene synthesis, was similar to those in Arabidopsis, rice and cassava (Fig. 5b). Two tandem repeat blocks were also found in the TPS-b subfamily (Fig. 5c). On the other hand, we found that many stress-related and defense-related cis-elements were in the promoter region of the ADH gene family (Fig. 5e). To confirm the classification of TPS family proteins in passion fruit, we constructed a phylogenetic tree by aligning the TPS proteins of passion and *Arabidopsis thaliana*^43^, and 21 PeTPSs belonged to the TPS-a subfamily, 16 belonged to the TPS-b subfamily, and three belonged to the TPS-f subfamily (Supplementary Fig. 17), which can also help elucidate the functions of TPS gene members in different subfamilies. Expression level analysis showed that the expression level of *PeTPS2/3/4/24* increased gradually with fruit development, which is consistent with the increasing trend of terpene metabolite contents (Fig. 5g). Additionally, one IDI, four MTS, five CCD, one FPS, one GGPS, one GPS, and two HDR genes were also identified. Although the number of members of these gene families was small, their expression levels changed substantially with fruit development; for example, the expression levels of *PeHDR1*, *PeCDD1*, and *PeMTS1/4* increased gradually with fruit development. These genes, especially the ACX, ADH, ALDH, and HPL genes, may play an important role in the metabolism of terpenoids.

## Discussion

Passion fruit, which is one of the most consumed fruits globally, is widely cultivated in tropical and subtropical areas. However, genetic research on this plant is currently hampered by the lack of a reference genome, which is due to the large size and high heterozygosity of the passion fruit genome (Supplementary Table 2). To elucidate the genetic system and evolution of Passifloraceae, we sequenced the genome of passion fruit, providing new insights and genomic resources for breeding. We report a high-quality chromosome-scale genome assembly of passion fruit, with a contig N50 of 3.1 Mb and assignment of 98.92% (1327.18 Mb) of the assembly to nine pseudochromosomes. The quality of this reference genome is much higher than that of related species, such as castor bean, with a scaffold N50 ~0.56 Mb^40^, as well as some recently sequenced fruit genomes, such as kiwifruit^44^ and star fruit^45^. The high quality of our assembly can be attributed to the use of the unique combination of Nanopore sequencing^46,47^ with chromosome-scale scaffolding via Hi-C^48^. The passion fruit genome sequence provides an important resource for future molecular breeding and evolutionary studies.

We found that passion fruit diverged after Brassicaceae and before Euphorbiaceae (Fig. 2a, d), which is consistent with the results of a molecular phylogenetic analysis based on the chloroplast genome^41,49^. *Ks* analysis of the passion fruit genome showed that an ancient WGD event and a modern WGD event occurred 65 MYA and 12 MYA, respectively. This explains why the passion fruit genome is much larger than those of *Ricinus communis*^40^ and *Manihot esculenta*^50^. It also suggests that WGD events are the most important determinants of plant genome size variation^51^. This genomic information for passion fruit will help clarify the evolutionary processes in Passifloraceae species and contribute to improving the understanding of the physiological and morphological diversity of Passifloraceae species.

As a medicinal and edible plant, passion fruit contains various biologically active substances. The metabolic and synthetic pathways of these substances are unclear. The genomic, transcriptomic, and metabonomic data provided new insights into the unique biosynthetic processes of passion fruit, showing that ‘metabolic pathways’ and ‘secondary metabolic pathways’ were the main pathways used to produce these active substances. The O2PLS model combined with gene expression level analysis identified nine candidate genes, of which four functionally annotated genes were *GDP-fucose Transporter 1-like*, *Tetratricopeptide repeat protein 33*, *protein NETWORKED 4B isoform X1*, and *Golgin Subfamily A member 6-like protein 22*. Metabolome analysis showed that esters, alcohols, aldehydes, and terpenes were the main volatile metabolites in passion fruit, and these volatile compound peak areas increased during ripeness, which was consistent with previous research results^52^. Among the passion fruit VOCs, esters, alcohols, and aldehydes account for a large proportion, and fatty acids (FAs) are regarded as the main precursors of ester-, alcohol-, and aldehyde volatiles^53–55^. FA-derived volatile organic compounds also make significant contributions to the flavor of many fruits and vegetables, including banana^56^, apple^57^, strawberry^58^, melon^59^, and tomato^55^. FAs are metabolized by β-oxidative enzymes, including LOX, HPL, ADH, AAT, and others. In addition, terpenes are important members of passion fruit VOCs, including linalool, (E) - 4,8-dimethylona-1,3,7-triene 3-buten-2-one, terpinen-4-ol, beta-ionone, and 3-carene. Terpenes are biosynthesized from the 5-carbon isoprenoid precursor isopentenyl-diphosphate (IDP) and its isomer dimethylallyl diphosphate (DMADP)^60^, and TPS enzymes then convert them into structurally diverse volatile monoterpenes and sesquiterpenes or semivolatile and nonvolatile diterpenes^61^. Terpenes are also abundant in many plants, such as the foliage of Eucalyptus^62^, tea tree^63^, coriander^64^ and carrot^61^, providing the characteristic smell of these plants. In this work, we analyzed key genes in the fatty acid and terpene metabolic pathways, and 13 and eight important gene families were identified in passion fruit, respectively. Interestingly, we found that the expression levels of *ACX13/14/15/20, ADH13/26/33, ALDH1/4/21, HPL4/6, FAD13/50/52/53/55, PeLOX5/18*, *PePDC3*, *PeFAE6*, *PeFAH2*, *PeCYP7/13*, *PeEHL13/15*, *PeACP-AP1/5/6/7*, and *PeAAT3* in the fatty acid pathway were relatively high and changed with fruit development, and that those of *PeTPS2/3/4/24, PeHDR1*, *PeCDD1*, and *PeMTS1/4* in the terpene synthesis pathway were relatively high and changed with fruit development, suggesting that these play important roles in VOC synthesis^43,65^. Notably, we found that both the ADH and TPS gene families have a total of 41 members in the passion fruit genome, tandem duplication was found to contribute to passion fruit ADH and TPS gene family expansion, and the expression patterns of ADH and TPS are different during fruit development and ripening. A phylogenetic tree generated by aligning the TPS proteins among passion and *Arabidopsis thaliana* revealed that 21 PeTPSs belong to the TPS-a subfamily, 16 belong to the TPS-b subfamily, and three belong to the TPS-f subfamily. The TPS-a subfamily consists of sesquiterpene and diterpene synthases, the TPS-b subfamily consists of monoterpene synthases, and the TPS-f subfamily consists of linalool synthases^43^. The classification of the TPS gene family was consistent with that of terpenes detected in the metabolome. Among the ADH and TPS gene families, the expression levels of *PeADH13/26/33* and *PeTPS2/3/4/24* increased gradually with fruit development, which is consistent with the increasing trend of ester and terpene metabolite contents. The same results were reported in studies of mango^66,67^ and pear^68^. These results indicated that these genes may play an important role in passion fruit flavor biosynthesis. Fruit flavor is an important index that reflects the quality of fruit and can attract consumers and enhance market competitiveness. Unfortunately, as a consequence of linkage with nearby genes, selection for alleles of genes associated with larger fruits altered metabolite profiles, altered fruit metabolite content and had strong negative effects on fruit flavor^69^. Passion fruit is characterized by a variety of fruit flavors; therefore, it is of great significance to study flavor metabolites and their related genes and to select and retain them in future breeding work.

In summary, the high-quality reference genome combined with transcriptome and metabolome analysis provided insights into the genome evolution and flavor synthesis of passion fruit. The data from this study also offer valuable resources for genetic studies and the improvement of passion fruit, including genome-assisted breeding of novel cultivars with desired traits and exceptional economic fitness.

## Supplementary information

Supplementary Figure 1-17

Supplementary Tables 1-23
